# Prediction of Residual Curing Capacity of Melamine-Formaldehyde Resins at an Early Stage of Synthesis by In-Line FTIR Spectroscopy

**DOI:** 10.3390/polym13152541

**Published:** 2021-07-31

**Authors:** Regina Seidl, Stephanie Weiss, Rudolf W. Kessler, Waltraud Kessler, Edith M. Zikulnig-Rusch, Andreas Kandelbauer

**Affiliations:** 1 Kompetenzzentrum Holz GmbH, Altenberger Straße 69, 4040 Linz, Austria, c/o W3C, Klagenfurter Straße 87–89, 9300 St. Veit an der Glan, Austria; r.seidl@wood-kplus.at (R.S.); s.weiss@wood-kplus.at (S.W.); e.zikulnig-rusch@wood-kplus.at (E.M.Z.-R.); 2 Department of Material Science and Process Engineering, Institute of Wood Technology and Renewable Materials, BOKU-University of Natural Resources and Life Sciences, Konrad Lorenz Strasse 24, 3430 Tulln, Austria; 3 Kessler ProData GmbH, 72762 Reutlingen, Germany; rudolf.kessler@reutlingen-university.de (R.W.K.); waltraud.kessler@reutlingen-university.de (W.K.); 4 Center for Process Analysis & Technology (PA&T), School of Applied Chemistry, Reutlingen University, Alteburgstrasse 150, 72762 Reutlingen, Germany; 5 Reutlingen Research Institute (RRI), Reutlingen University, Alteburgstrasse 150, 72762 Reutlingen, Germany

**Keywords:** melamine formaldehyde (MF), inline spectroscopy, process analytics, batch modelling, decorative laminates

## Abstract

Melamine-formaldehyde (MF) resins are widely used as surface finishes for engineered wood-based panels in decorative laminates. Since no additional glue is applied in lamination, the overall residual curing capacity of MF resins is of great technological importance. Residual curing capacity is measured by differential scanning calorimetry (DSC) as the exothermic curing enthalpy integral of the liquid resin. After resin synthesis is completed, the resulting pre-polymer has a defined chemical structure with a corresponding residual curing capacity. Predicting the residual curing capacity of a resin batch already at an early stage during synthesis would enable corrective measures to be taken by making adjustments while synthesis is still in progress. Thereby, discarding faulty batches could be avoided. Here, by using a batch modelling approach, it is demonstrated how quantitative predictions of MF residual curing capacity can be derived from inline Fourier Transform infrared (FTIR) spectra recorded during resin synthesis using partial least squares regression. Not only is there a strong correlation (R^2^ = 0.89) between the infrared spectra measured at the end of MF resin synthesis and the residual curing capacity. The inline reaction spectra obtained already at the point of complete dissolution of melamine upon methylolation during the initial stage of resin synthesis are also well suited for predicting final curing performance of the resin. Based on these IR spectra, a valid regression model (R^2^ = 0.85) can be established using information obtained at a very early stage of MF resin synthesis.

## 1. Introduction

Melamine-formaldehyde (MF) resins have been widely used in the wood-processing industry as glues, binders and coatings for many decades due to their outstanding properties [1]. Exceptional resistance against hydrolysis and chemicals as well as remarkable mechanical properties such as high scratch and abrasion resistance make MF resins indispensable for most engineered wood products [2,3,4,5].

**Figure 1:**
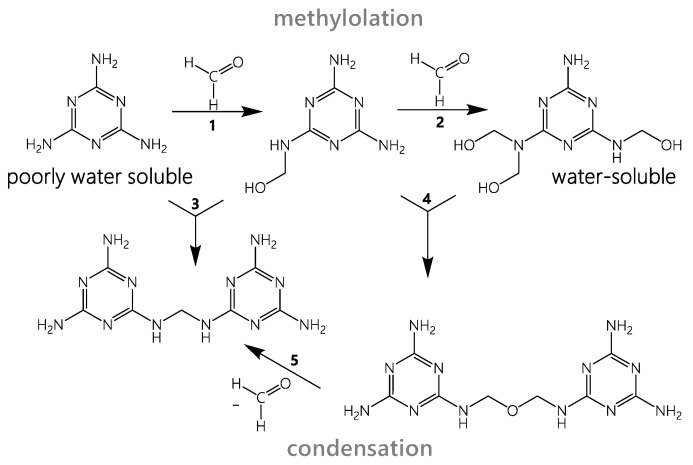
General reactions taking place in MF resins. Methylolation: Water insoluble melamine reacts with formaldehyde to water-soluble methylolated species (1, 2). Condensation: Formation of methylene bridges (3), formation of methylene ether bridges (4) and rearrangement under formaldehyde release (5). Modified from [12].

Since the 1950s, much research has been conducted to understand melamine-formaldehyde resins and to develop MF resins for special applications. The fundamentals are already well understood [6,7]. MF resin synthesis can be roughly divided into two phases (Figure 1). (1) The first phase is methylolation of melamine by formaldehyde and concomitant complete dissolution of melamine. This leads to completely transparent solutions at the so-called “clear point” (CP). (2) The second phase is condensation, which leads to oligomeric pre-polymers, that are ultimately cured to give highly cross-linked thermoset networks via subsequent molding or hot pressing processes. The synthesis at the pre-polymer stage is interrupted when a pre-defined water tolerance level (usually 350%) of the resin is reached at the so-called “turbidity point” (TP) [8,9,10,11].

Methylolation as well as methylene-ether and ether bridges formation take place under alkaline or acidic conditions. They cannot be clearly separated from one another [13], [14] since methylols participate in condensation reactions as soon as they are formed. Thus, the reaction mixture becomes very complex already at an early stage of conversion and contains a multitude of isomers and network fragments. The relative abundance of these chemical species will determine the further course of network formation and, ultimately, the final resin properties [15,16]. Therefore, one should be interested in following the molecular changes taking place during resin formation by appropriate means of process analytical technology (PAT) and relating them to resin performance [17]. While already well-established in the chemical, biotechnological and pharmaceutical industries [18], the benefits of real-time and in-line process analytical approaches are not yet widely exploited by the wood processing industry. However, in order to cope with the ever increasing demands imposed by global competition, it is all the more important that these rather low-added-value production processes, in particular, be better understood at a deeper level.

**Figure 2:**
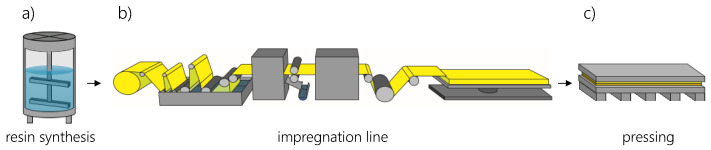
Three-step production process of decorative laminates. (**a**) Resin synthesis. (**b**) Impregna-tion line. (**c**) Pressing of the impregnated paper on a substrate (e.g., particle board). In the first step, the impregnating resin is synthesized. In the subsequent impregnation channel, raw paper is passed through the impregnating resin bath and then dried. In the third step, the dry impregnated paper is processed on a substrate material.

The present work is dedicated to the first step in the process sequence for producing decorative laminates (Figure 2), i.e., resin synthesis. As shown earlier [19], condensation of the resin does not progress much during impregnation and drying (second process step, Figure 2b) under the process conditions typically applied in the industry [19]. It was also shown earlier [20,21,22] that the curing conditions applied in process step 3 (Figure 2c) have a significant effect on the final properties of cured MF resin films. Laminate properties can be tuned by applying appropriate hardening schemes to one and the same liquid resin [20,21,22]. However, this is only possible within certain limits and both the processing behavior and the performance in solid state of a resin are mainly determined by the applied synthesis conditions and, in particular, by the melamine-to-formaldehyde ratio [23]. Resins produced within an initial pH range of 7.9 and 12.1, and a range of melamine-to-formaldehyde M:F ratio from 1:1.5 to 1:4.5, show very different property profiles in terms of viscosity, penetration into paper, and residual curing capacity, although these resins all displayed the same water tolerance (as determined by titration with water) and have the same polarity (as determined by contact angle measurements) [23]. These results illustrate that the properties of the resin are to a very large degree already determined during the synthesis by the reaction conditions. While normally, technological resin properties are only tested on the final resin after synthesis is finished, it would be desirable to predict the main performance criteria already at an earlier stage during synthesis. This would offer the opportunity to anticipate unfavorable batch properties at a stage where taking corrective action is still possible and would allow means of feed-forward process control to be implemented to avoid waste production. To date, no means of spectroscopic inline control have been implemented in industrial MF resin synthesis with the aim of quantitatively predicting technological quality parameters of the resulting impregnation formulations on a routine basis. Here, it is shown that by multivariate calibration of inline infrared spectra recorded during resin synthesis, the residual curing capacity of an MF resin can be predicted as early as directly after complete dissolution of the reactants in the reaction medium at a very early stage of resin synthesis.

## 2. Materials and Methods

### 2.1. Materials

Paraformaldehyde (>99%), melamine (>99%) and sodium hydroxide (NaOH) were purchased from Carl Roth GmbH & Co KG (Karlsruhe, Germany). All chemicals were used as received.

### 2.2. Resin Preparation

**Figure 3:**
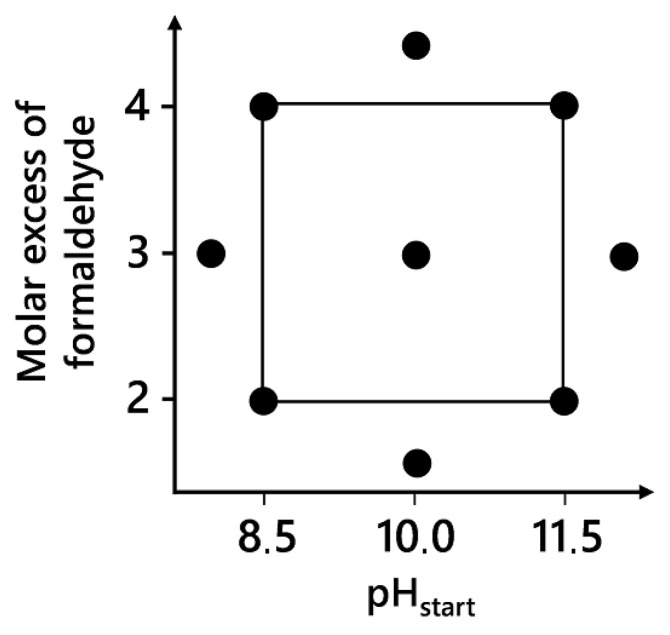
Design space with schematic representation of three types of experiments: four center point replications, four factorial design points and four axial points.

Melamine-formaldehyde resins were synthesized basically according to the process described by Pizzi [5,23]. The general approach was as follows. According to the experimental design (see Figure 3 and Section 2.3) the calculated amount of paraformaldehyde and melamine powder was added to deionized water. Then, the pH value was adjusted using diluted aqueous NaOH. The reaction mixture was heated to 95 °C. The pH decreased slowly over time during the reaction. The water tolerance of the reaction mixture was tested at regular time intervals by withdrawing small samples and diluting them with deionized water. At a water tolerance of 350% the so-called turbidity point (TP) is reached and resin synthesis was stopped.

### 2.3. Experimental Design

Figure 3 shows how the experiments were distributed in the design space. A central composite design (CCD) for two factors was used [24]. The melamine to formaldehyde ratio was varied in a range from 1.5 to 4.5. At the beginning of the synthesis, the initial pH value was adjusted in the range from 7.9 to 12.4. In total, 12 MF resin syntheses were performed. The detailed settings are given in Table 1. The effects of melamine to formaldehyde ratio (factor 1) and initial pH of the reaction mixture (factor 2) on the resin synthesis up to the defined water tolerance level was investigated by in-situ FTIR.

### 2.4. Differential Scanning Calorimetry (DSC)

Thermograms were recorded with a DSC 3 differential scanning calorimeter by Mettler Toledo (Greifensee, Switzerland). An amount of 5 mg of resin was weighed into gold-plated high-pressure stainless steel crucibles (30 µL). High-pressure crucibles were used to suppress evaporation of water and other volatile substances during heating. For the heating program, a linear temperature ramp from 30 to 220 °C with a heating rate of 5 °C min^−1^ was used. The STARe 15.00a software package (Mettler Toledo, Greifensee, Switzerland) was used to determine the enthalpy integral of the exothermic curing signal in order to determine the residual curing capacity of the resins. All measurements were carried out three times.

### 2.5. Inline Infrared Spectroscopic Analysis

**Scheme 1:**
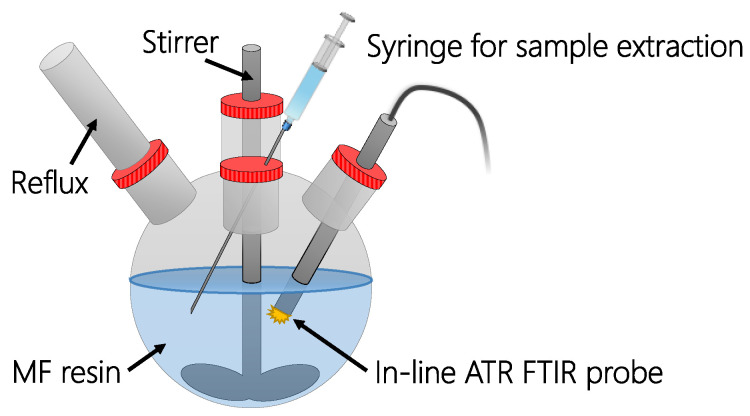
Experimental setup for inline spectroscopic measurements.

To monitor the progress of MF resin synthesis, a ReactIR 15 (Mettler Toledo, Greifensee, Switzerland) equipped with a liquid nitrogen cooled MCT detector was used. An in-situ probe based on attenuated total reflection (ATR) FTIR technology was inserted directly in the reaction flask. The experimental setup for inline spectroscopic measurements is depicted in Scheme 1. Every minute, a spectrum of the reaction mixture was acquired in the spectral range from 3000–650 cm^−1^ with a scan rate of 128 and a resolution of 4 cm^−1^. The background spectrum was measured against air. Data recording was carried out by the software package iC IR 7.0 (Mettler Toledo, Columbus, OH, USA).

### 2.6. Multivariate Data Analysis

Multivariate data analysis of the IR spectra was performed using the Unscrambler^®^ X 10.5 software package (CAMO Software AS, Oslo, Norway). As pre-treatment, the spectra were mean centered and normalized (unit vector normalization). The spectral range of the fingerprint region from 1750–750 cm^−1^ was analyzed by principal component analysis (PCA). The principal components (PCs) were determined using the NIPALS-algorithm. Leverage correction was used as validation [25,26]. Correlation of spectral and thermal data was carried out by partial least squares regression (PLS-R). The x-variable (input data) were the normalized and mean centered MIR spectra (1750–750 cm^−1^). The y-variable (target parameter) was the residual curing capacity as measured by DSC. The multivariate regression algorithm Kernel was used. As test matrix for validation the center point replicates were used [27].

## 3. Results and Discussion

### 3.1. Spectral Time Course of MF Resin Synthesis and Infrared Band Assignment

**Figure 4:**
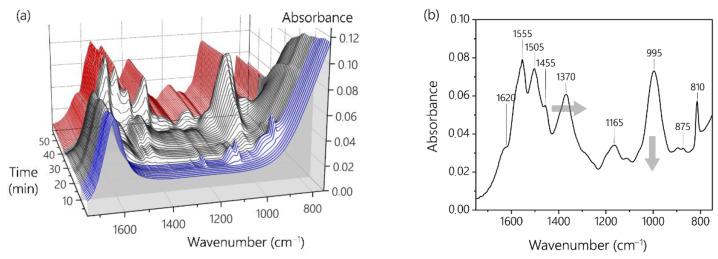
Infrared spectra in the spectral range from 1750 to 750 cm^−1^. (**a**) 3D plot of IR spectra during an entire MF resin synthesis procedure. The initial phase of filling the reactor is colored in blue. The subsequent phase of dissolving melamine via methylolation until a clear reaction mixture is obtained is colored in black. The subsequent phase of chemical changes occurring in homogenous solution (resin condensation) is colored in red. The spectral time course of MF resin with an M:F ratio of 3 and a starting condition of pH 10 (center point) is shown. (**b**) Last spectrum of this time series at the end of the synthesis giving the characteristic spectral fingerprint of the resin. The arrows indicate the directions of the most significant changes during the condensation phase.

The progress of the MF resin synthesis was monitored by in-line FTIR spectroscopy. Figure 4a shows a typical time course of MIR spectra in the region 1750–750 cm^−1^ for an entire MF resin synthesis including the filling of the reactor with distilled water, paraformaldehyde and melamine (phase 1, spectra colored in blue), dissolution of the reactants in water (phase 2, spectra colored in black), and condensation in homogenous solution from the point of clarification (CP) to the turbidity point (TP) (phase 3, spectra colored in red). The first spectrum (at minute 0) shows the absorbance bands of water. Upon dissolution of the chemical compounds, the absorbance bands increase and gradually change as the chemical reaction takes place. The last spectrum (taken at min 57) of the spectral time course shows the characteristic absorption pattern of the finished resin at the TP. For clarity, this final spectrum is shown separately in Figure 4b along with the most important characteristic wavenumbers.

In the spectral region from 1750 to 750 cm^−1^, melamine shows the characteristic absorbance bands found in melamine formaldehyde resins (Figure 4b). The following interpretation of infrared bands is based on peak assignments given in [20,21,28,29,30]. The absorbance peak at 815 cm^−1^ is assigned to the triazine ring. The C–O stretching vibrations of the methylol functionality are found at 995 cm^−1^ [30]. In the spectral region from 1200–1150 cm^−1^ several absorbance peaks overlap: C–N stretching, C–O–C and C–N–C bending vibrations from methylene and ether bridges and primary aromatic amines (NH_2_) show twisting and rocking vibration. At 1370 cm^−1^, a C–N stretching vibration stemming from melamine occurs [28]. The position of this peak depends on the substituent linked to the vibrating functional group. It shifts depending on the electronegativity or the mass of the attached groups [29]. From 1600–1400 cm^−1^, several peaks overlap to a great extent. The peak at 1455 cm^−1^ is assigned to C–H vibrations and in-plane O–H deformation vibrations. The absorbance found at 1555 cm^−1^ is due to triazine ring vibrations. The peak at 1620 cm^−1^ is assigned to N–H deformation vibration of –NH_2_.

The two arrows in Figure 4b indicate the direction of the chemical changes during the condensation phase in homogenous solution. The absorbance peak at 1370 cm^−1^ shifts towards smaller wavenumbers. The absorbance signal at 995 cm^−1^ decreases as the methylol moieties react to methylene-ether and methylene bridges.

### 3.2. Process Trajectories of MF Resin Synthesis

#### 3.2.1. Time Course of Entire MF Resin Synthesis

In a first step the entire reaction course for all 12 resin syntheses (844 individual spectra) was evaluated within one data set. Since the variance within the spectroscopic signals during the last phase of MF synthesis is comparatively small, subsequently, in a second step, only the chemical changes taking place during reaction in homogenous phase are analyzed, i.e., the condensation phase up to the TP.

**Figure 5:**
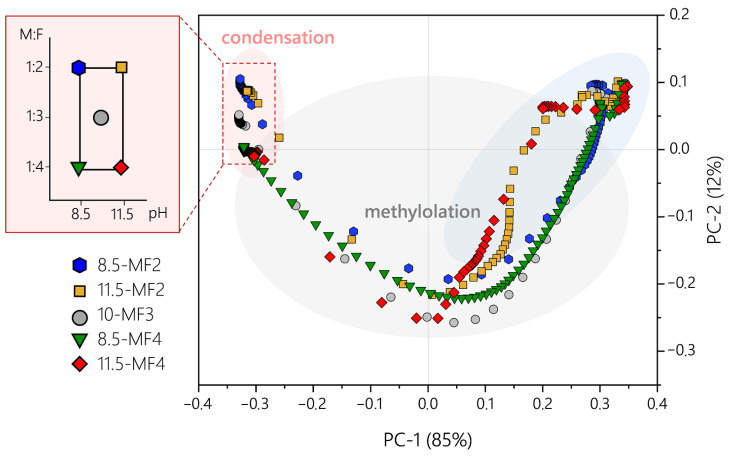
Process trajectories for PCA. Score plot (PC1 vs. PC2) of the PCA analysis of the entire synthetic procedure for five of the prepared MF resins. The score plot shows the process trajectories of the factorial experiments (triangular, rhombic, hexagon and square symbols) and center point (circle) settings of the MF resin syntheses. The reaction phase is indicated by blue (phase 1: reactor filling), grey (phase 2: dissolution/methylolation heterogeneous reaction system) and red (phase 3: condensation in homogenous reaction medium) shadows.

The first principle component (PC1) is associated with the main spectral changes taking place over the complete time course of MF resin synthesis. PC1 explains 85% of the total spectral variance. The remainder of variations in the spectral data set is explained by PC2 and PC3, which account for 12% and 2%, respectively. Hence, 99% of the variance in the spectra during the dissolution, methylolation and condensation process can be explained with the first three principal components. The score plot given in Figure 5 shows the score values of PC1 and PC2 for each spectrum, respectively. The time series of the scores (i.e., every spectrum is represented by one data point) gives the “process trajectory” [31]. The concept of process trajectories allows graphical comparison of the differently synthetized MF resins.

The different phases of the MF cooking procedure are indicated by different background shadings. Phase 1: reactor filling phase is assigned a color shading blue. Phase 2: heterogeneous reactant dissolution/methylolation phase is assigned the color shading grey. Phase 3: homogenous condensation phase is assigned a color shading red. The colored symbols for the different process trajectories given represent the different experimental settings used for the synthesis of the respective MF resin. The insert on the left-hand-side of Figure 5 shows a scheme illustrating the experimental design points facilitating the assignment of the reaction conditions to the process trajectories.

The process trajectories given in Figure 5 (PC1 vs. PC2 of the PCA analysis for the entire reaction procedure) show a U-shaped progress with reaction time. For the condensation phase, the variance among the recorded infrared spectra is very small compared to the overall changes taking place during all three reaction phases. Therefore, in Figure 5 the distance between the points representing a FTIR spectrum is hardly noticeable. To better see the changes taking place specifically in the homogenous reaction mixture from the point of clarification (CP) to the turbidity point (TP, end of reaction), a more detailed analysis focusing on the condensation phase is presented in Section 3.2.2.

**Figure 6:**
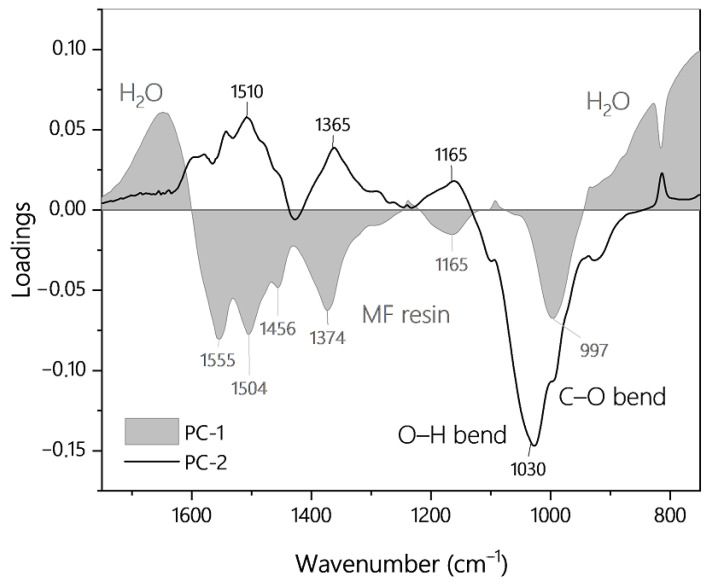
Loadings plots of PC1 and PC2 of the PCA analysis of the entire synthesis procedure. For better distinction of the two PCs, PC1 has a light grey background and PC2 is a solid line.

The loadings plot in Figure 6 shows at which wavenumber regions the original data show the largest deviations from the average infrared spectrum. Hence, the loadings plot can be used to identify the most important chemical changes that are taking place. For interpretation and correlation with spectral information, the score and loadings plots need to be combined as follows. Spectra with positive score values show above-average absorbance values in the regions where the loadings plot shows positive values. In contrast, they have below-average absorbance values in wavenumber regions with negative loading values. Spectra with negative score values have above-average absorbance in regions with negative loadings values and below-average absorbance in regions of positive loadings values.

PC1 explains 85% of the spectral variance of the entire MF resin synthesis. All MF resin syntheses show analogous behavior on PC1. With increasing reaction time, the score values move from positive to negative PC1 score values. The loadings for PC1 show an increase in absorbance in the spectral region from 1600–950 cm^−1^ with reaction time and a decrease in absorbance in the spectral regions around 1650 cm^−1^ and < 940 cm^−1^. The latter wavenumbers are associated with O–H vibrations of water. In the spectral region from 1600–950 cm^−1^ the peaks at 1555, 1505, 1455, 1375, 1165, and 995 cm^−1^ are characteristic absorbance peaks for MF resins (see Figure 4). The in-plane and out-of-plane stretching vibrations of the triazine ring dominate the spectral regions from 1580–1520 cm^−1^ and 1450–1350 cm^−1^ [29]. The absorbance at 995 cm^−1^ is assigned to the C–O stretching vibration of the methylol functionality. Moreover, in the spectral region from 1200–1150 cm^−1^, several overlapping absorbance peaks are present (see Section 3.1).

This time course reflects very well the physical and chemical changes associated with the experimental procedure and the general chemistry of MF resin formation described in the literature [32]. At the beginning of the synthesis, water and undissolved paraformaldehyde and melamine are present in the reaction mixture. Characteristic MF resin absorption peaks rise as methylolated melamines appear due to chemical dissolution of melamine via methylolation. The process continues as the reaction mixture is heated to reaction temperature. The absorbance peaks of water systematically decrease due the increasing contribution of the MF resin absorbance peaks to the total intensity (spectral dilution effect).

PC2 explains 12% of the spectral variance of the entire MF resin synthesis. With increasing reaction times, the spectra show positive and negative score values. Starting from positive PC2 score values during filling the data points proceed towards negative score values during dissolution and continue further towards positive values at the end of the synthesis. The loadings plots show that the main differences in the spectral information on PC2 is due to changes in the absorbance peak at 1030 cm^−1^. As paraformaldehyde and water combine to methylene glycol (HO–CH_2_–OH), characteristic peaks at 1023 and 922 cm^−1^ due to bending vibrations of O–H and C–H are expected at the beginning of the dissolution process [29]. At the minimum score value of PC2, the highest content of formalin is present in the reaction mixture. With increasing reaction time the score values proceed in direction of positive PC2 as the formalin reacts with melamine. The methylolated melamine dissolves and is represented by the positive peaks in the loadings plot at 1510, 1365, and 1165 cm^−1^.

Towards the end of resin synthesis, a specific pattern of the score values can be discerned. This pattern corresponds well with the distribution of the experimental settings of the central composite design in the design space (see the data points located within red rectangle in Figure 5). Samples with high amounts of formaldehyde (M:F ratio 1:4) show the lowest score values for PC2. Low PC2 values correspond to a higher content of formalin (O–H bending of methylene glycol) and higher degree of methylolation (C–O stretching vibrations in –CH_2_–OH). Center point (M:F ratio 1:3) and low formaldehyde resins (M:F ratio 1:2) show correspondingly higher PC2 values.

**Figure 7:**
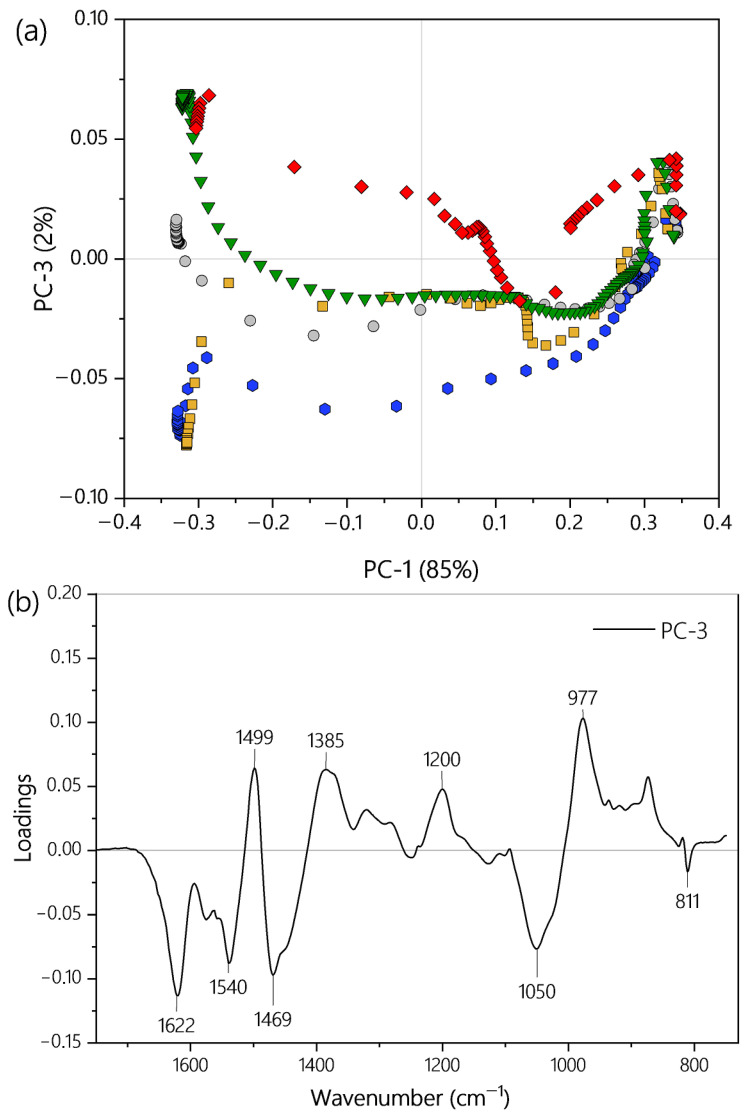
Process trajectories for PCA. (**a**) Score plot (PC1 vs. PC3) of the PCA analysis of the entire synthetic procedure for five of the prepared MF resins. The score plot shows the process trajectories of the factorial experiments (triangular, rhombic, hexagon and square symbols) and center point (circle) settings of the MF resin syntheses. The reaction phase is indicated by blue (phase 1: reactor filling), grey (phase 2: dissolution/methylolation heterogeneous reaction system) and red (phase 3: condensation in homogenous reaction medium) shadows. (**b**) Loadings plots of PC3 of the PCA analysis of the entire synthesis procedure.

PC3 explains 2% of the spectral variance observed within the entire MF resin synthesis. Starting at the point of total dissolution and towards the end of the synthesis the score values (see Figure 7a) form a cluster in the process trajectories based on PC1 versus PC3. The corresponding loadings plot, giving the major spectral changes summarized by PC3, is given in Figure 7b.

The loadings plot shows that spectra with positive PC3 scores have higher than average absorbance values at the following spectral regions: the increased absorbance band with a wavenumber at 980 cm^−1^, which is assigned to the C–O stretching vibrations of methylol functionalities –CH_2_–OH, and the increased absorbance at 1200 cm^−1^, which is related to C–N stretching vibrations and the asymmetric C–O–C stretching vibration in –CH_2_–O–CH_2_–.

The absorbance band at around 1380 cm^−1^ is particularly interesting since it is assigned to an X sensitive stretching vibration of aromatic amines [29]. The vibration frequency of the CAr–X bond shifts depending on the mass or electronegativity of the substituent. Based on the relative order of electron withdrawing properties and molar masses of the functional groups involved, in MF resins, methylol groups should be expected to appear at the relatively highest frequencies followed by methylene ether and methylene bridges. For the increasing extent of bridge formation in the condensation phase, it should therefore be expected that this peak shifts towards smaller wavenumbers as methylol functionalities are consumed and transformed into bridges vibrating at smaller wavenumbers. However, the “starting wavenumber” of a specific resin depends on the M:F ratio of that resin. High M:F ratio resins show higher absorption at 1380 cm^−1^ as a higher number of methylolated species occurs due to the higher formaldehyde content.

At around 1500 cm^−1^ several C–H vibrations, O–H and N–H deformation vibrations occur. This fits the overall picture that resins with high M:F ratio have a higher degree of methylolation and show an above average content of ether bridges.

In contrast, spectra with negative PC3 values show higher absorbance signals at 810 and 1540 cm^−1^, which are absorbance bands characteristic of the triazine ring. The absorbance at 1620 cm^−1^ can be assigned to N–H deformation vibrations of –NH_2_. The peak at 1050 cm^−1^ cannot clearly be assigned to one functional group. It is unlikely that this absorption occurs only due to the O–H bending mode of formaldehyde (see PC2) since corresponding characteristic peaks are missing. Spectra of melamine powder shows an absorbance peak at 1020 cm^−1^ due to the triazine ring deformation vibrations and –NH_2_ rocking, which is in better accordance to the already assigned peaks of PC3 [28]. This absorbance peak is also reported by Mircescu et al. [33]. Resins with high melamine to formaldehyde ratio have a higher degree of methylolation and a higher number of bridges. Conversely, in resins with low formaldehyde content, the absorbance bands of the triazine ring and absorption peaks of the amino group vibrations predominate.

Summing up, in the multivariate analysis of the spectral time course over the entire synthesis procedure, PC1 summarizes the most general changes in absorbance properties of the forming MF pre-polymers that can be assigned to dissolution, temperature increase and reaction time. PC2 mainly contains information on formalin and formation of methylolated species. The formation of chemical bridges during the condensation phase is mainly covered by changes visible on PC3. Towards the end of the resin synthesis, the formation of point clusters of the score values is observed, which can be assigned to the different starting conditions in terms of different formaldehyde content (factor M:F ratio). Since the nature and distribution of bridged oligomeric species is of great interest for the properties of the final pre-polymer, the condensation phase in homogenous solution of the MF resin is of great interest. However, in the analysis so far, also covering the whole synthetic procedure including dissolution and methylolation, it can only be analyzed based on the third principal component which explains only 2% of total variance. Therefore, to achieve a more detailed and informative picture, in the next step, the multivariate analysis focused only on the chemical changes going on during this last stage of the synthesis and only those spectra were analyzed that were recorded in homogenous phase between the point of clarification (CP) and the turbidity point (TP).

#### 3.2.2. Time Course of the Condensation Reaction in Homogenous Medium (Phase 3 of MF Resin Synthesis)

**Figure 8:**
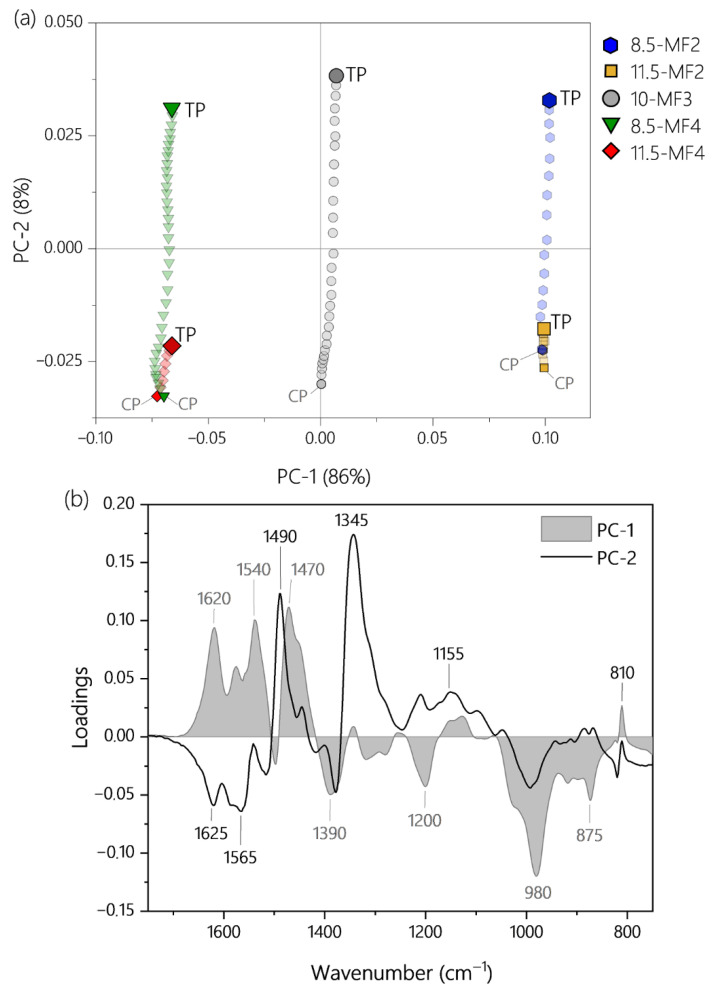
Process trajectories for PCA. (**a**) Score plot (PC1 vs. PC2) of the PCA analysis of the final condensation phase of resin synthesis in homogenous phase for five of the prepared MF resins. The score plot shows the process trajectories of the factorial experiments (triangular, rhombic, hexagon and square symbols) and center point (circle) settings of the MF resin syntheses. (**b**) Loadings plots of PC1 and PC2 of the PCA analysis of the reaction in homogenous phase procedure. For better distinction of the two PCs, PC1 has a light grey background and PC2 is a solid line.

In this subsection, only the reaction in homogenous solution, i.e., the condensation phase from the point of total dissolution up to the endpoint of the reaction, is analyzed. Only the spectra from CP to TP (see data points marked in red, Figure 8) are included in the principle component analysis.

The explained variances of the two principal components are 86% and 8% for PC1 and PC2, respectively. With the first two PC2 already 94% of the total spectral variance in the condensation phase can be described. In Figure 8a, the score values representing the FTIR spectra of the condensation phase are now clearly separated. The loadings plot for PC1 and PC2 is depicted in Figure 8b.

PC1 explains 86% of the spectral variance in the condensation phase. After the point of complete dissolution (clear point, CP), three clusters of data points stand out. On the left side of Figure 8a (PC1 score values <0) the data points representing the spectra of resins with high formaldehyde content (M:F ratio 1:4) form one group. Another group representing spectra of resins with an M:F ratio of 1:3 is located in the middle region of the PCA space (PC1 score vales ≈0) and a third point group representing spectra with low formaldehyde content (M:F ratio 1:2) can be seen on the right hand side of the PC space (PC1 score values > 0). The corresponding loadings of PC1 (Figure 8b) indicate that PC1 contains the same spectral information as PC3 of the first analysis (absorbance values mirrored). The peak assignment is given in a previous subsection (see interpretation of PC3 (2%), Section 3.2.1).

PC2 explains 8% of the spectral variance in the condensation phase. For this data set the score values of the MF resin spectra progress from negative to positive values on the PC2 axis. According to the loadings of PC2 (Figure 8b), the spectral patterns of the final resins at the turbidity point (located on the positive side of PC2 axis) have comparatively much higher absorbance values at 1345, 1155 and 1490 cm^−1^ than their counterparts at the point of clarification (CP). The absorbance at 1345 cm^−1^ is assigned to the C–X sensitive peak which shifts from 1385 to 1345 cm^−1^ as the reaction proceeds (due to formation of bridges). The absorbance maximum observed at 1155 cm^−1^ results from the formation of C–O–C vibrations of methylene-ether bridges. These trends are in accordance with the absorbance peaks associated with the negative loadings, which can be assigned to melamine (1565 cm^−1^), –NH_2_ (1625 cm^−1^) and methylol moieties (990 cm^−1^). The peak at 1490 cm^−1^ cannot clearly be assigned, since in this region C–H, O–H, and N–H vibrations overlap to a great extent. Nevertheless, it is most likely that this absorbance is due to C–H vibration since PC2 calculated in this second analysis corresponds to the oligomerization of MF resin.

Summing up, the final spectral patterns obtained with the studied set of prepared MF resins as visible in the score plot (Figure 8a) very much reflect the experimental conditions, i.e., the systematic variations in M:F ratio and the initial pH introduced to the MF resin synthesis. PC1 explains the main spectral variations taking place during the condensation phase in homogenous solution and very strongly reflects the range in applied melamine to formaldehyde ratio of the resins. PC2 contains mainly information associated with bridge formation and reflects the experimental variations introduced by settings of the initial pH value in the resin synthesis.

### 3.3. Predicting Residual Curing Capacity of MF Resin Using PLS-Regression

#### 3.3.1. PLS-Regression in the Spectral Range from 1750 to 750 cm^−1^

In this section, the quantitative prediction of MF residual curing capacity from FTIR spectra by multivariate regression is described. The residual curing capacity is of great importance for decorative laminates, as no additional adhesive is added in the final production step of pressing the impregnated paper on a substrate board. Therefore, a sufficient amount of residual curing capacity is crucial for the quality of the final product. Table 1 shows the experimental design and the enthalpy H (integral of the exothermic curing signal) of the resins as well as the mean value of the center point replicates. Instead of the single measured values, the mean value of the center point replicates is used for the calibration set in the PLS regression. The four center point replicates are used as validation data set (see Table 2). Which points were used for calibration (cal) und which were used for validation (val) is indicated in Table 1.

For the prediction of the residual curing capacity, the last spectra of MF resin synthesis at the endpoint TP were used as input data (in the spectral region from 1750 to 750 cm^−1^).

**Figure 9:**
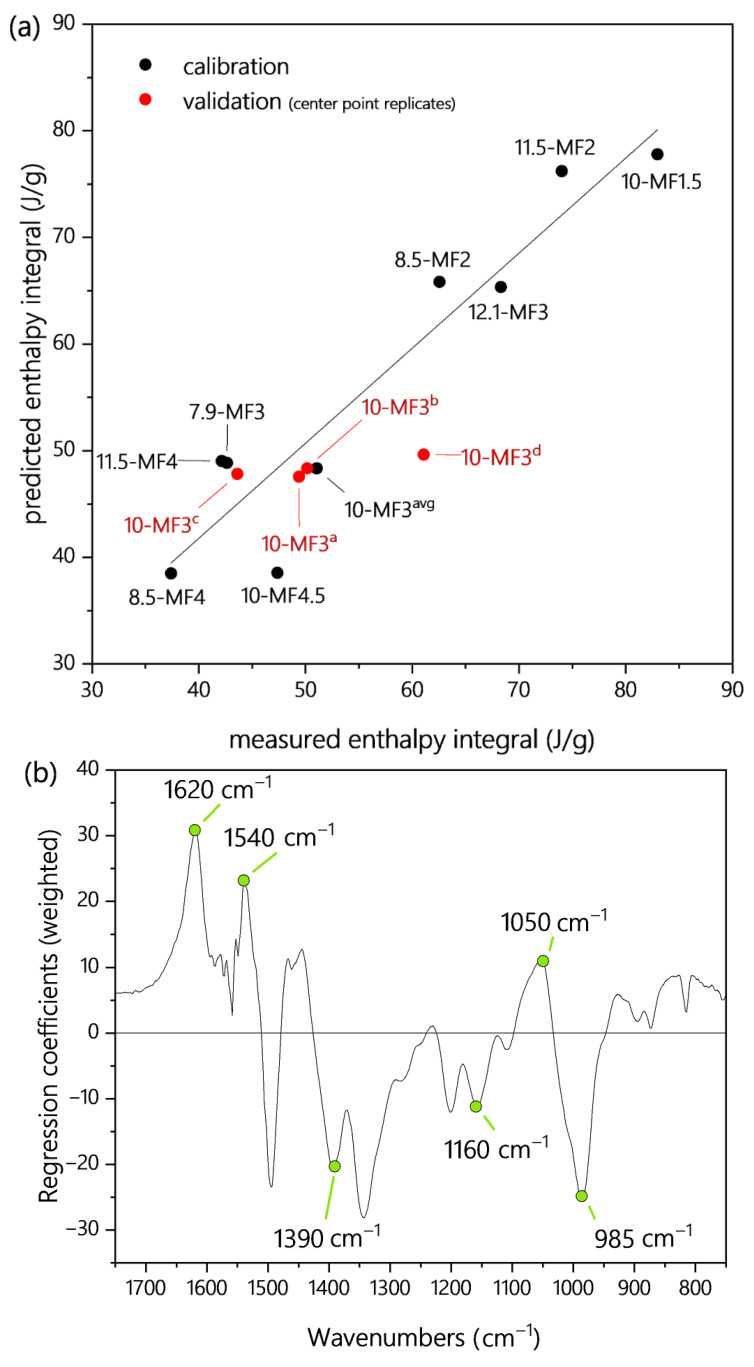

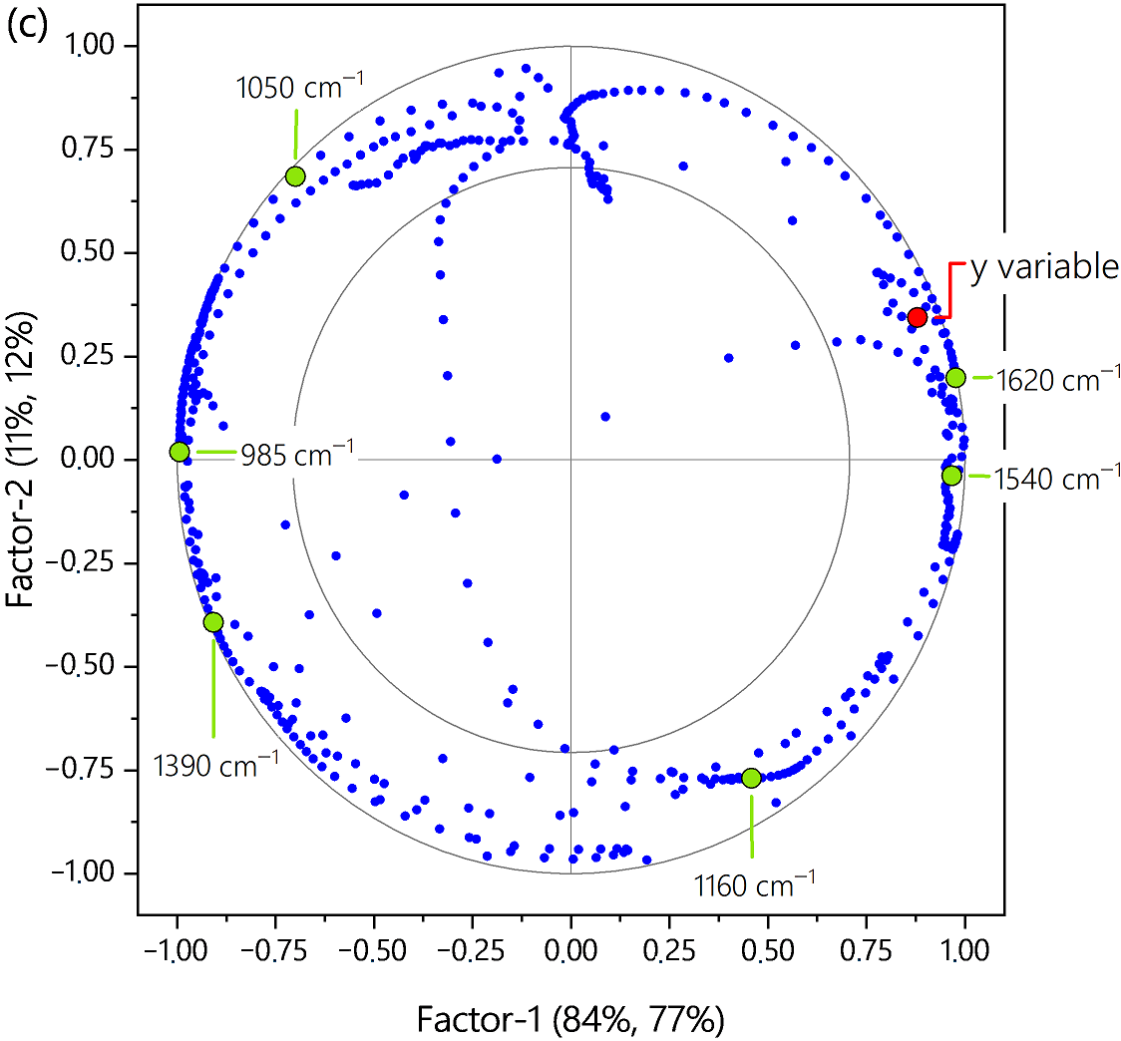
PLS model at TP for predicting residual curing capacity calculated with two factors. (**a**) Predicted vs. reference values for residual curing capacity. (**b**) Weighted regression coefficients for two factors vs. x-variables (fingerprint region 1750–750 cm^−1^). (**c**) Correlation loadings plot with outer-circle 100% explained variance, inner circle 50% explained variance, spectral input data (blue, green) and response residual curing capacity (red).

In Figure 9a the predicted residual curing capacity calculated with two factors (PLS-components) is shown versus the calibration standard (measurement values). The regression coefficients (see Figure 9b) indicate the importance of the following specific absorbance regions for the prediction of residual curing capacity: the wavenumbers: 985, 1050, 1160, 1390, 1540, 1620 cm^−1^ are most important for the PLS regression model. To display the relationship between all variables used in the calculation the correlation loadings (x and y) plot is shown in Figure 9c.

Figure 9a shows the values predicted by the regression equation and the measured reference values. Factor 1 explains 84% of spectral variance and 77% of the variance of enthalpy H. Factor 2 explains 11% of spectral variance and 12% of the variance of enthalpy H. For the two factor model the coefficient of determination (R^2^) of calibration is 0.89 and the root mean square error of calibration (RMSEC) is 4.98. The residuals were checked to make sure that for small and large predicted y variables the unexplained variance is in the same order of magnitude.

Figure 9b shows the weighted regression coefficients for the two factors regression model. Since all absorbance values and all response values y are positive, the interpretation is as follows. Higher absorption in the regions of positive regression coefficients is correlated with higher response values y. Therefore, the model predicts higher residual curing capacity for resins with high absorption at wavenumber regions around 1620, 1540 and 1050 cm^−1^. These absorption regions dominate in MF resins with low amount of formaldehyde (M:F 1:2) and are assigned to –NH_2_ and triazine ring vibrations. In contrast, higher absorption in the region of negative regression coefficients is associated with lower response values. Lower residual curing capacity is connected to higher absorbance at 1340, 1160 and 980 cm^−1^. These peaks are above-average in MF resins with high formaldehyde content (M:F 1:4). The peaks are assigned to methylene-ether bridges and also display a higher degree of methylolation, which is in accordance with the surplus of formaldehyde.

A lower degree of pre-condensation leads to a higher residual curing capacity and vice versa. This confirms earlier findings [23] and can be explained by the fact that resins with lower M:F ratios need to form less bridges during the synthesis to reach the same water tolerance as resins with high M:F ratio. MF resins with high formaldehyde content (M:F 1:4) show high content of methylol moieties (potential cross-linker), but due to the already rather advanced cross-linking state the rigidity of the network suppresses further bridge formation [21].

The correlation loadings plot in Figure 9c shows the spectral input data (spectral range from 1750 to 750 cm^−1^, blue and green). The response “residual curing capacity” is marked in red. Due to the information contributed by each wavenumber, the corresponding data point is positioned in the new coordinate system spanned by the PLS-components (factors). From the position of the response target value, it is evident that the information from both PLS-components is necessary to describe the residual curing capacity. Especially highlighted (green) are wavenumbers which are most important for the calibration and which are used for an outlook given in Section 3.3.2.

Table 2 shows the validation of the PLS regression model via center point replicates. Mean value, standard deviation (SD) and standard error of mean (SE) are given for the reference y value (enthalpy H) and the predicted y value containing the spectral information.

Although the mean values for y reference and y predicted are close together, clear differences are seen in variability and precision of the center point replicates. The standard deviation of enthalpy H is about 10 times larger than SD of y predicted (based on spectral data). The standard error of mean (sample size *n* = 4) is about eight times larger for the enthalpy H than for y predicted. This shows on the one hand the large variability in enthalpy H as measured via DSC. On the other hand, it illustrates the superior precision of spectroscopic data. In the literature the major cause for the uncertainty associated with DSC measurements is attributed to the temperature gradient between crucible and thermocouple or the contact between crucible and sample holder [34,35] but it may also have to do with the general properties of samples with high water content and correspondingly only weak heat flow signal. Due to the variation in heat enthalpy values, in the present study the mean value of the center point replicates was used for calibrating the PLS model. This was carried out because as the number of experimental values increases, the average result will tend to closer reflect the true value [36].

With the spectral region from 1750–750 cm^−1^ at TP it was demonstrated, that a PLS regression of spectral data and the residual curing capacity of the final MF resin is very well possible. However, even more interestingly, it was found that a robust regression model (R^2^ = 0.85, RMSEC = 5.87) can also be found even at a much earlier stage of the reaction, i.e., already at the clear point (CP) during resin synthesis. Analogous to the analysis discussed above, the same spectral region was used for all MF resins at the respective point of complete dissolution to predict the enthalpy integral. The results are summarized in Table 3 and show that predictions are equally well possible and it is not necessary to wait until synthesis is completed in order to anticipate the curing properties. For an early prediction of the residual curing capacity, the characteristic point of total dissolution (CP) is already suitable. Earlier stages of synthesis, however, seem to be not suitable since at before the point of clarification not yet all chemicals are present in solution and therefore, cannot be detected by FTIR.

#### 3.3.2. PLS-Regression Using a Reduced Data Set and Concept for a Low-Cost Process Spectrometer

The conception of a low-cost process analyzer, as an alternative to an expensive MIR spectrometer, requires a model of comparable quality with reduced input data (which is based on only a comparatively small number of wavenumbers (x-variables)) [37].

The most important wavenumbers for successful PLS modelling as identified from using the full spectral range (region from 1750 to 750 cm^−1^, Figure 9) are: 1620, 1540, 1390, 1160, 1050, 985 cm^−1^. Since this characteristic wavenumbers describe the response variable best, it was attempted to model the target response solely based on these wavelengths and omitting all other input information. The quality parameters characterizing the resulting models are presented in Table 3.

For all input data sets the coefficient of determination R^2^ was >82%. Compared to the original model calculated with the entire spectral range, all models based on only a reduced number of wavenumbers show comparable quality. This can be explained by the fact that only the relevant x-variables are used for the modelling. By neglecting non relevant information from the input data, it was possible to achieve a comparable model with a significantly reduced input data set. However, in order to identify this set of most relevant wavelength it was necessary to analyze the entire fingerprint region in the first step.

## 4. Conclusions

In this study, inline FTIR spectroscopic data recorded during the synthesis of melamine formaldehyde resins were analyzed using the multivariate data analysis methods Principal Component Analysis (PCA) and Partial Least Squares Regression (PLS-R). PCA scores were used to track and visualize the chemical changes taking place during the reaction as “process trajectories”. The spectral variance observed during the condensation phase when resin oligomers were formed in homogenous solution allowed for the assignment of the major chemical changes taking place to the two process factors varied in the experimental design in terms of functional group vibrations. Using PLS regression, the MIR spectra of the differently synthesized MF resins were correlated quantitatively to the residual curing capacity of the final resin. Mathematical models to predict the thermal curing behavior in dependence of the process factors M:F ratio and initial reaction pH were derived at two different stages of the synthesis: (1) at the end of the synthesis (after reaching the turbidity point, TP), and (2) at an early stage of the synthesis (after completion of melamine dissolution at the clarification point, CP). For model building, different sets of spectral wavenumbers were used: either (1) the full spectral range from 1750-750 cm^−1^, or (2) only six selected wavenumbers (1620, 1540, 1390, 1160, 1050, 985 cm^−1^), or (3) a further reduced data set containing only three selected wavenumbers (1620, 1540, 985 cm^−1^) were used. It was shown that good predictive models for residual curing capacity can be obtained already at an early stage of resin synthesis as soon as a homogenous reaction mixture has formed. This opens up the possibility of feed forward process control by following the synthesis process in real-time using low-cost infrared spectroscopy instrumentation which focuses only on the essential wavenumbers. If deviations from the expected process trajectories are detected, the immediate consequences on resin quality (curing behavior) can be quantitatively estimated and corrective measures can be undertaken in time to avoid faulty batch production.

## Figures and Tables

**Table 1:** Experimental design, response values for residual curing capacity (enthalpy H) with mean value of center point replicates and usage in model (cal, calibration; val, validation).

Resin	Factors		Response	Use in PLS Model
	pH_start_	M:F Ratio	Enthalpy H (J/g)	
8.5-MF2	8.5	1:2.0	62.56	cal
11.5-MF2	11.5	1:2.0	74.01	cal
8.5-MF4	8.5	1:4.0	37.41	cal
11.5-MF4	11.5	1:4.0	42.15	cal
7.9-MF3	7.9	1:3.0	42.65	cal
12.1-MF3	12.1	1:3.0	68.30	cal
10-MF1.5	10.0	1:1.5	82.97	cal
10-MF4.5	10.0	1:4.5	47.37	cal
10-MF3 ^a^	10.0	1:3.0	50.16	val
10-MF3 ^b^	10.0	1:3.0	49.39	val
10-MF3 ^c^	10.0	1:3.0	43.62	val
10-MF3 ^d^	10.0	1:3.0	61.08	val
10-MF3 ^avg^	10.0	1:3.0	51.06	cal

^a, b, c, d^ measured center point replicates; ^avg^ calculated mean value of center point replicates.

**Table 2:** Mean value, standard deviation (SD) and standard error of mean (SE) of center point replicates.

	y Reference (Enthalpy H)	y Predicted (Spectral Information)
Mean value	51.06	48.34
SD	6.31	0.80
SE	3.16	0.40

**Table 3:** Statistics of PLS models calculated with two factors for spectral information obtained at two different stages of the synthesis, endpoint (TP) and the clear point (CP).

Stage of Synthesis Used	Model Based On	Wavenumbers (cm^−1^)	R^2^_cal_	RMSEC
TP	Entire spectral range	1750–750	0.89	4.98
TP	6 single wavenumbers	1620, 1540, 1390, 1160, 1050, 985	0.92	4.20
TP	3 single wavenumbers	1620, 1540, 985	0.82	6.86
CP	Entire spectral range	1750–750	0.85	5.87
CP	6 single wavenumbers	1620, 1540, 1390, 1160, 1050, 985	0.85	5.77
CP	3 single wavenumbers	1620, 1540, 985	0.90	5.17

## Data Availability

Data are available upon request from the authors.
